# Identifying the hidden burden of allergic rhinitis (AR) in community pharmacy: a global phenomenon

**DOI:** 10.1186/s40733-017-0036-z

**Published:** 2017-11-21

**Authors:** Rachel Tan, Biljana Cvetkovski, Vicky Kritikos, David Price, Kwok Yan, Pete Smith, Sinthia Bosnic-Anticevich

**Affiliations:** 1 Quality Use of Respiratory Medicines Group, Woolcock Institute, University of Sydney, Sydney, Australia; 2 Academic Primary Care, University of Aberdeen, Aberdeen, UK; 3 Observational and Pragmatic Research Institute Pte Ltd, Singapore, Singapore; 4 Royal Prince Alfred Hospital, Sydney, Australia; 5 Clinical Medicine, Griffith University, Southport, QLD Australia; 6 Sydney Local Health District, Sydney, Australia

**Keywords:** Allergic rhinitis, Consult pharmacists, Self-manage, Suboptimal management

## Abstract

**Background:**

Patients with allergic rhinitis often trivialise their condition, self-manage inappropriately, and would benefit from health care intervention. The primary point of health care contact for these self-managing allergic rhinitis patients is the community pharmacy. With the majority of allergic rhinitis treatments being available for purchase over the counter, without health care professional contact, we know little about how the patients self-manage. This study aims to identify the burden of allergic rhinitis in the community pharmacy and to identify key opportunity for intervention.

**Methods:**

Pharmacy customers, who purchased nasal treatment in a community pharmacy, were approached with a research-administered questionnaire that collected data on medical history, symptoms and products purchased for the treatment of nasal symptoms.

**Results:**

Of the 296 participants, 69.9% self-managed with over-the-counter medications; with 68% experiencing allergic rhinitis symptoms and only 44.3% of this subgroup had a doctor’s diagnosis. Nasal congestion (73.6%) was most commonly experienced and oral antihistamines were most commonly purchased (44.3%), indicating a pattern of suboptimal management. A third of participants (36.5%) experienced moderate-severe symptoms, persistently, which impacted on their daily living. Medication selection was mainly based on pharmacy customers’ perceptions of medication effectiveness (47.6%).

**Conclusion:**

A majority of participants that self-selected over-the-counter medications have symptoms consistent with allergic rhinitis, with almost half not having received a diagnosis. Medication purchasing patterns suggest that sub-optimal therapeutic decisions made by participants, even when they are experiencing significant symptoms. This study uncovers the hidden burden of allergic rhinitis in the community pharmacy and a missed opportunity to intervene and refer if necessary. Patients need to be guided through appropriate treatment as this study showed that many should be referred to a medical practitioner.

**Electronic supplementary material:**

The online version of this article (10.1186/s40733-017-0036-z) contains supplementary material, which is available to authorized users.

## Background

Allergic Rhinitis(AR) is a chronic respiratory condition characterised by sneezing, itching, rhinorrhoea and nasal congestion [1], induced by IgE-mediated inflammation in the nose, in response to allergens [1]. Although AR shares similar nasal symptoms with non-AR or rhinosinusitis [2], it is a distinct chronic disease of the upper airways, which is not a minor ailment. AR currently affects 10–30% of the world’s population [3] and the prevalence is still increasing [1]. It presents a significant health burden especially when it is uncontrolled [1], impairing an individual’s day-to-day activities, causing sleep disturbance resulting in daytime fatigue and affecting work productivity [1, 4, 5]. The ramifications of poorly controlled AR extend into other disease states, most notably asthma, where the likelihood of exacerbations or flare-ups is elevated [1]. Given that approximately 50% of people with asthma have poorly controlled disease [6], the importance of treating coexisting AR is paramount [1].

Differential diagnosis of AR from other forms of rhinitis is not easily defined as AR rarely presents in isolation [7]. This complicates the management of AR [2] yet many sufferers are still self-selecting in the community pharmacy [8]. To optimise self-selection of AR medication, patients would ideally have received a diagnosis from a health care professional (HCP) and are able to determine the appropriateness of medication from the manufacturer’s written information [9]. In theory this may seem feasible, however many with nasal symptoms do not seek diagnosis or advice [10]. Given that the management of AR is not straightforward, it requires proper medical guidance for self-management, in order to ensure that optimise treatment outcomes are achieved [1].

In Australia, many of the AR treatments are ‘pharmacy medicines’, ‘Schedule 2’, stored on open shelves and available over the counter (OTC), without prescription and without the requirement of pharmacist intervention [11]. Patients seeking to purchase AR treatments in the community pharmacy do so without consulting a pharmacist [8] although pharmacists are accessible [12], they are underutilised [13]. Patients tend to perceive their illness to be a ‘minor’ ailment, which they can easily, manage themselves [10]. The ease of medication accessibility [14], their lack of awareness of importance of appropriate treatment [15] and their low expectation of the pharmacist [16], could be the potential reasons for patient interactions with pharmacists to be less than ideal [17].

In order to optimise the management of AR in the community, it is important to understand to what extent people with AR self manage and why. With AR treatments being available over the counter in pharmacies, the burden is hidden and hence we are unable to identify opportunities to intervene and optimise management. Therefore, this study aims to identify patients who self-select and self manage their AR within a community pharmacy setting, in order to gain an appreciation of the burden on pharmacy and also identify opportunities where pharmacists can intervene to deliver health care interventions and optimise AR management.

## Methods

This research took the form of a cross-sectional observational study of pharmacy customers purchasing treatments for nasal symptom(s) from community pharmacies during July–September 2015 (Australian spring) and April–June 2016 (Australian autumn). All participants provided informed consent prior to enrolling in the study.

### Participants and setting

Community pharmacies within the Sydney metropolitan area, which had expressed an interest in research or pharmacy services, were approached to participate in this research. The sample of pharmacists approached to participate were strategically chosen to ensure that pharmacies covered a spectrum of socio-demographic locations. Participation in the study involved giving permission for the researcher to conduct a survey with customers who fulfilled the inclusion criteria and did not violate the exclusion criteria, within the community pharmacy setting.

**Fig. 1:**
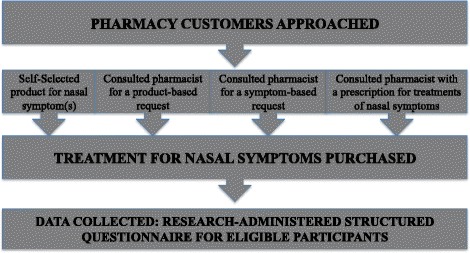
Study design overview

The inclusion criteria for study participants were as follows: pharmacy customers who visited the pharmacy and independently self-selected OTC treatment(s) for nasal symptom(s) without pharmacist advice; or consulted the pharmacist for advice regarding treatment(s) for nasal symptom(s); or consulted the pharmacist requesting a specific treatment for nasal symptom(s); or presented a prescription for treatment of nasal symptom(s) (Fig. 1). Pharmacy customers were included in the study if they were purchasing treatments for themselves or for other family members and had the knowledge to answer the questions on behalf of that family member. Pharmacy customers were excluded from the study if they were purchasing treatments other than for nasal symptoms or were unable to answer questions relating to the purchase of the product when purchasing for another family member or someone else.

### Survey

**Fig. 2:**
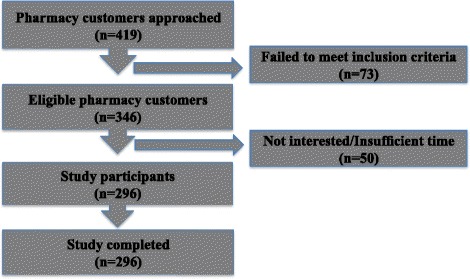
Inclusion of participants with nasal symptoms in the study (n = 296)

A researcher-administered structured questionnaire was developed based on published literature, the research and practice expertise of the investigators and the framework of current international guidelines for the management of AR(1) (Additional file 1). The study questionnaire covered three domains: i) demographic characteristics (age, gender, who the medication was for, method of product selection, diagnosis), ii) nasal (clinical) symptoms for which the product was being purchased (nature, frequency, duration and severity of symptoms, and triggers) and iii) management of nasal symptoms (treatment purchased) (Fig. 2). Based on the literature it was expected that a cohort of patients would be seeking treatment for AR, despite not previously being diagnosed by a doctor, therefore, in order to describe the study population as accurately as possible, three clinical experts evaluated the potential presence of AR based on patient reported nasal symptoms and triggers identified [18]. With the data collected the expert clinical panel determined whether presenting nasal symptoms were related to AR, viral infections such as a common cold/flu, or other known or unknown causes. Based on the reported prevalence of self-selection medication for AR, a sample of 200 AR patients was required to achieve a representative sample [19].

### Data analysis

All data collected were de-identified. Data were analysed using SPSS (IBM® SPSS® Statistics) Version 22. Descriptive statistics were used for continuous and categorical variables.

## Results

Each pharmacy that was approached to participate in the study, agreed to do so. A total of 8 pharmacies were recruited and of the 419 pharmacy customers approached by the researcher, 296 met the inclusion criteria and agreed to participate. Reasons for non-participating are summarised in Fig. 2. Of the 296 participants, 17.9% (53/296) were purchasing nasal treatments for other family members and could provide a full account of the nature of the condition.

Table 1 summarises the clinical characteristics of participants self-selected against those who consulted a pharmacist. Sixty-three percent (187/296) of the participants had a doctor’s diagnosis for their presenting nasal symptoms. A total of 67.9% (201/296) were classified as having AR (Table 1), of which 65.2% (131/201) had a doctor’s diagnosis and 35.3% (71/201) were identified by the expert clinical panel. Others (14.2%, 42/296) included a combination of non-allergic rhinitis, sinusitis, postnasal drip and those who are unidentifiable (8.4%, 25/296) included a range of nasal symptoms for which a definitive cause of symptoms could not be determined without further referral to a medical practitioner.

**Table 1:** Demographics and clinical characteristics of participants (*n* = 296)

Variable	All participants (n = 296)	Self-selected OTC treatment(s) (*n* = 206)	Consulted with the Pharmacist (*n* = 90)		
			Product based request (*n* = 42)	Symptom based request (*n* = 30)	Prescribed treatment (*n* = 18)
Gender					
Female	197 (66.6%)	142 (68.9%)	21 (50.0%)	22 (73.3%)	12 (66.7%)
Male	99 (33.4%)	64 (31.1%)	21 (50.0%)	8 (26.7%)	6 (33.3%)
Age group					
< 18	20 (6.8%)	15 (7.3%)	3 (7.1%)	0 (0%)	2 (11.1%)
18–39	102 (34.5%)	72 (35.0%)	12 (28.6%)	8 (26.7%)	10 (55.6%)
> 40	174 (58.8%)	119 (57.8%)	27 (64.3%)	22 (73.3%)	6 (33.3%)
Differential diagnosis					
AR	201 (67.9%)	139 (67.5%)	31 (73.8%)	16 (53.3%)	15 (83.3%)
Cold/Flu	28 (9.5%)	22 (10.7%)	2 (4.8%)	3 (10.0%)	1 (5.6%)
Other	42 (14.2%)	31 (15.0%)	4 (9.5%)	5 (16.7%)	2 (11.1%)
Unidentifiable	25 (8.5%)	14 (6.8%)	5 (11.9%)	6 (20.0%)	0 (0%)
Symptoms					
Sneezing	198 (66.9%)	139 (67.5%)	28 (66.7%)	18 (60.0%)	13 (72.2%)
Rhinorrhoea	212 (71.6%)	147 (71.4%)	31 (73.8%)	20 (66.7%)	14 (77.8%)
Nasal Congestion	218 (73.6%)	149 (72.3%)	31 (73.8%)	23 (76.7%)	15 (83.8%)
Itchy Nose	81 (27.4%)	64 (31.1%)	9 (21.4%)	3 (10.0%)	5 (27.8%)
Itchy Eyes	151 (51.0%)	110 (53.4%)	22 (52.4%)	12 (40.0%)	7 (38.9%)
Itchy Ears/palate	55 (18.6%)	41 (19.9%)	6 (14.3%)	4 (13.3%)	4 (22.2%)
Wheeze	37 (12.5%)	32 (15.5%)	2 (4.8%)	0 (0%)	3 (16.7%)
Headache	28 (9.5%)	22 (10.7%)	2 (4.8%)	2 (6.7%)	2 (11.1%)
Fever	6 (2.0%)	5 (2.4%)	0 (0%)	1 (3.3%)	0 (0%)
Duration of Symptoms					
Intermittent	144 (48.7%)	96 (46.9%)	22 (52.4%)	16 (53.5%)	10 (55.6%)
Persistent	152 (51.4%)	110 (53.4%)	20 (47.5%)	14 (46.7%)	8 (44.4%)
Severity of symptoms					
Mild	31 (10.5%)	25 (12.1%)	4 (9.5%)	2 (6.7%)	0 (0%)
Moderate-Severe	265 (89.5%)	181 (90.5%)	38 (90.5%)	28 (93.3%)	18 (100%)
Impact of symptoms on QOL					
Daily activities	111 (37.5%)	68 (33.0%)	19 (45.2%)	12 (40.0%)	12 (66.7%)
Performance	27 (9.1%)	15 (73%)	4 (9.5%)	3 (10.0%)	5 (27.8%)
Sleep	79 (26.7%)	58 (28.2%)	10 (23.8%)	5 (16.7%)	6 (33.3%)
None	118 (39.9%)	96 (46.6%)	10 (23.8%)	11 (36.7%)	1 (5.6%)
Triggers of symptoms					
Identified	180 (60.8%)	130 (63.1%)	27 (64.3%)	11 (36.7%)	12 (66.7%)
Onset period of symptoms					
Seasonal	149 (50.3%)	105 (51.0%)	27 (64.3%)	9 (30.0%)	8 (44.4%)
Year Round	67 (22.6%)	55 (26.7%)	3 (7.1%)	7 (23.3%)	2 (11.1%)

**Fig. 3:**
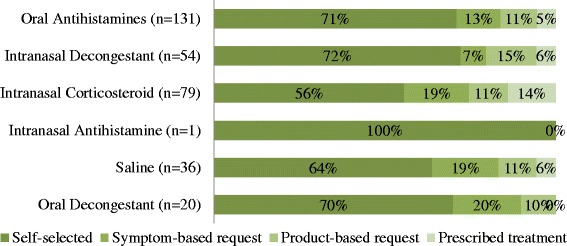
Proportion of products purchased by various management methods (*n* = 296) (Percentage of participants)

Rhinorrhoea and nasal congestion were most commonly experienced (Table 1). A majority of symptoms were experienced to a moderate-severe extent (Table 1), persistently for the majority of participants with AR (55.7%, 112/201) and intermittently for the majority of the participants with cold/flu (71.4%, 20/28) (Fig. 3). The majority of participants who reported wheezing also had AR (84.2%, 32/38). When participants were asked “*what symptoms is this product(s) being used to treat?”,* 51.7% (153/296) reported only one symptom. On further questioning, 48.3% (143/296) reported more than one symptom; with 57.7% (116/201) of the AR participants underreporting the number of symptoms experienced.

Of the participants that reported an impact of symptoms on their quality of life (QOL), the most recognisable impact was on daily activities (Table 1). Fourteen percent (42/296) of participants reported impact on more than one aspect of their QOL.

The majority of the participants identified a trigger that worsened their symptoms (Table 1), and 23.3% (69/296) identified more than one. For the AR participants, the most common outdoor allergen reported was pollen (54.7%, 110/201) and indoor allergen was house dust mite (34.3%, 69/201). More than half of the AR participants attributed their onset of symptoms to a seasonal basis (Table 1).

**Fig. 4:**
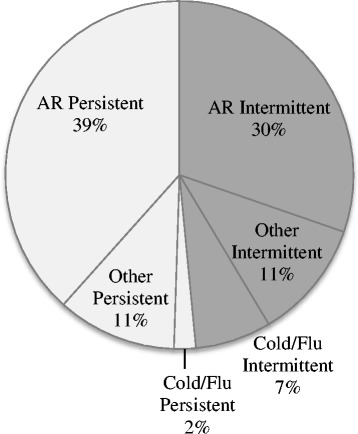
Proportion of participants experiencing intermittent or persistent symptoms (n = 296). *Other participants = alternative diagnosis, non-allergic rhinitis or unable to determine diagnosis

Table 2 summarises the various medications purchased by participants. Oral antihistamines and intranasal corticosteroids were most commonly purchased to treat AR (89% and 77% respectively). The majority of participants with cold/flu purchased intranasal decongestants or saline (Table 2). Figure 4 compares the proportion of medications purchased via self-selection or consulted with the pharmacist, which includes medications with a prescription. There was statistically significant difference between the proportion of participants who purchase an intranasal corticosteroid when comparing participants who consulted with the pharmacist, compared with those who self-selected (38.9% vs 21.4% respectively, χ^2^ = 9.837, *p* = 0.003) but there was no statistically significant difference between the proportion of participants who purchase an oral antihistamine when comparing participants who consulted with the pharmacist, compared with those who self-selected (42.2% vs 43.7% respectively, χ^2^ = 0.055, *p* = 0.899).

**Table 2:** Therapeutic classes of treatments purchased by participants (n = 296)

Therapeutic classes	All participants (n = 296)	Self-selected OTC treatment(s) (n = 206)	Consulted with the pharmacist (n = 90)		
			Product-based request (n = 42)	Symptom-based request (n = 30)	Prescribed treatment (n = 18)
Purchased Medication					
Oral Antihistamine	131 (44.3%)	93 (45.1%)	17 (40.5%)	14 (46.7%)	7 (38.9%)
Intranasal Decongestant	54 (18.2%)	39 (18.9%)	4 (9.5%)	8 (26.7%)	3 (16.7%)
Intranasal Corticosteroids	79 (26.7%)	44 (21.4%)	15 (35.7%)	9 (30.0%)	11 (61.1%)
Intranasal Antihistamine	1 (0.3%)	1 (0.5%)	0 (0%)	0 (0%)	0 (0%)
Saline	36 (12.2%)	23 (11.2%)	7 (16.7%)	4 (13.3%)	2 (11.1%)
Oral Decongestant	20 (6.8%)	14 (6.8%)	4 (9.5%)	2 (6.7%)	0 (0%)

Forty-eight percent (141/296) of the participants self-selected their medications based on their perception of medication effectiveness. The next common reason was based on HCP’s recommendation (13.5%, 40/296), followed closely by interaction with a pharmacist (15.5%, 46/296). There were also other reasons (16.9%, 56/296) which included price concern, instructions written on the package, recommended by family or friend, advertisement, ingredient preference and seeking others of the same class of drugs.

## Discussion

This study identified that there is a substantial, hidden burden of AR in community pharmacy. Just under three quarters of participants self-selected their product, based on the patient’s perception of what they felt was most effective. Amongst those who consulted the pharmacist, almost half of them had a product request; only a third a symptom request and a fifth of them came with a prescription. More than half the participants who come into the pharmacy to treat nasal symptoms had AR and a striking 56% of these patients did not have a HCP diagnosis of AR; even though the majority were experiencing moderate-severe symptoms, on a persistent basis. The most common symptom experienced was nasal congestion, however the most common therapeutic class of treatment purchased was oral antihistamine, which does not address congestion. These results highlight the need for pharmacist intervention in selecting medication for the treatment of nasal symptoms.

AR was the most common condition experienced by participants seeking treatment for nasal symptoms. Only half the participants had a diagnosis, a trend that has existed for years (7, 20). While there are guidelines that base the diagnosis of AR on symptoms history with allergy confirmation via a skin prick test or radioallergosorben (RAST) test [1]; patients are not consulting the HCP, therefore these guidelines should be tailored to real life health behaviours. Since this study showed that with appropriate questioning, a pharmacist is able to identify patients with a high likelihood of having AR, pharmacy intervention, in the form of risk assessment or a screen tool are required. In fact, the process used to identify and clarify the cause of nasal symptoms in this study suggest that implementation of such tools would be feasible for use in the community pharmacy setting.

Pharmacists are also in a position to identify patients that do require referral. While a majority, either through previous diagnosis or pharmacist questioning were able to have the cause of their nasal symptoms identified, 25 participants required immediate referral to the doctor, for symptoms which could not be attributed to obvious causes. This highlights the importance of pharmacist’s engagement with patients, even for what are perceived to be trivial symptoms. Despite the common prevalence of nasal symptoms, they can be presented in a complex way and be indicative of a more complex condition. Pharmacists need to have the opportunity to identify those patients who need immediate referral to a doctor.

In addition to participants’ poor perception of the ‘seriousness’ of their AR (given the severity of symptoms experienced by majority of the participant), they also seem to have a superficial perception of the extent of symptoms experienced. Patients seemed to focus on one key symptom, often fail to mention the range of symptoms they were experiencing except upon in-depth questioning. This has important implications for treatment option as with the combination of patients underestimating their condition [20] and poorly perceiving their symptoms [21], there is a potential for delayed diagnosis. Therefore, this indicates the importance the role of community pharmacists in the management of AR.

This study also suggests that patients may overestimate their self-management ability. They recognised their primary symptoms and noted an impact on their day-to-day living, yet did not see the need to seek medical attention, even when the impact on their day-to-day living was significant. This indicates that there is a disconnection between patient experiences of symptoms and their health behaviours, resulting in the inappropriate selection of products. Participants relied on their perception of medication effectiveness, which is consistent with the Health Belief Model (HBM) [22], rather than what is clinically known to be the optimal treatment. HBM states that self-management behaviour is driven by patient’s perceived susceptibility (the threat of getting a disease before they take the next step in management) perceived severity of the condition (realisation of the severity of disease before consulting a health care professional), perceived benefits of treatment and perceived barriers or potential adverse effects resulting from their actions [22]. This re-iterates the importance of HCPs’ advice which enables patients to fully comprehend the correct use of medication [23]. It could also provide a solution to the trend observed in the management of AR i.e. for patients to keep searching for more effective medications for AR, as they are not satisfied with their current treatment [24].

These results have clinical implications especially for participants with AR. This study showed the feasibility of a pharmacist-implemented tool to identify the cause of nasal symptoms within a community pharmacy setting. There is a critical necessity for pharmacists to identify patients with chronic upper airway disease so that they can receive appropriate treatment. This is particularly important for the management of AR. This study showed that intranasal corticosteroids were preferred over oral antihistamine to treat AR, for participants who consulted with the pharmacist. This treatment selection is in accordance with the international guideline – Allergic Rhinitis and its impact on Asthma (ARIA) guideline [25]. ARIA guidelines developed over the past 20 years have improved the care of AR patients [25]. Participants who obtained professional advice from a pharmacist had a higher chance of choosing the recommended medication for their condition. Pharmacist are well placed to identify the symptoms of AR and recommend appropriate OTC treatment, and therefore important in many areas of intervention in AR.

While a potential limitation of this study is the non-random nature of the selection of pharmacies into this study, this descriptive study provides a significant evidence to target further exploration into the way in which pharmacists can better engage with patients purchasing OTC products for the treatment of AR. Patients are clearly at risk of suboptimal or inappropriate management based on patient self-perceived effectiveness and self-selection of products for AR, this can have significant impact on co-morbidities, in particular asthma.

## Conclusion

In conclusion, this research confirms that the level of self-management of AR in the community pharmacy is high, that pharmacist engagement is low and that there is a significant missed opportunity to ensure that optimal management of AR with OTC products is enabled. This study indicates that as a result of patients driving their own treatment, that burden of AR in community pharmacy is not only high, but also hidden. This is particularly important when we consider that a majority of these patients are attempting to self-treat AR without a diagnosis or HCP input, even when they may have a co-existing asthma. In order to ensure referral to a doctor is made in a timely manner for patients with AR and subsequent appropriate OTC treatment is being selected, pharmacists need to be up skilled in this area of care, and they need to be provided with tools e.g. screening tools, risk assessment tools. Future research needs to explore ways in which guideline-driven clinical pathways can be implemented and evaluated in the community pharmacy setting. It may be that this is potentially one of the most effective ways of controlling AR-relate comorbidities, such as asthma in the future.

## Additional file

Additional file 1: Researcher administered survey (DOCX 21 kb)
