# Supplementary material for: Identifying the hidden burden of allergic rhinitis (AR) in community pharmacy: a global phenomenon
Source: Asthma Res Pract. 2017 Nov 21;3:8. doi: 10.1186/s40733-017-0036-z (PMC5696909; doi:10.1186/s40733-017-0036-z)
Supplement: Additional file 1: — Researcher administered survey (DOCX 21 kb) [file 40733_2017_36_MOESM1_ESM.docx]

**Additional file 1**

| **Researcher administered survey:**  **The management of nasal symptoms: PATIENT PRODUCT SELECTION FORM** | | | | | | | | | |
| --- | --- | --- | --- | --- | --- | --- | --- | --- | --- |
| **Do you consent to participate in this study?** | | | | - Yes | | | | - No | |
| **PRODUCT(S):** | | | | | | | | | |
| **Product(s) selected: _____________________________**  **What are you taking it for? ______________________**  **Why did you choose this product(s)?**   - Effective 🡺 **□** Compared to others of the same class - Price/Advertisement/Catalogue/What’s on the box - Recommended, Who? ________________________ - Other: | | **Who for?** | | | - Self | | | - Other | |
|  |  | **Age:** | | | | - <18 | - 18-39 | | - >40 |
|  |  | **Gender:** | | | | - M | - F | | - Pregnant |
| **DIAGNOSIS:** | | | | | | | | | |
| **Have you spoken to your doctor about this?**  If ‘yes’, what was diagnosed and/or recommended? | | | - Yes, GP/Specialist | | | | | - No | |
| If ‘no’, did you speak to your pharmacist?  If ‘yes’, what was recommended/advised? | | | - Yes | | | | | - No | |
| **Has anyone shown(spray)/explained(tablet) how to use this?** If ‘yes’, please specify: | | | - Yes, GP/Pharmacist | | | | | - No | |
| **MEDICATION HISTORY:** | | | | | | | | | |
| **Have you tried anything in the past for these condition?**  **Did it work for you?** | | | - Yes,______________ - Yes | | | | | - No - No | |
| **Are you using anything else for your condition?**  **Do you use a puffer?** | | | - Yes, ______________ - Yes, _______________ | | | | | - No - No | |
| **SYMPTOM(S):** | | | | | | | | | |
| **What symptom(s) is this product(s) being used to treat?** | | | | | | | | | |
| **Do you also have?**   \| **Symptoms** \| \| \| --- \| --- \| \| - Sneezing \| - Watery Eyes \| \| - Itchiness   Nose/Eyes/Ears/Palate \| - Mucus   Clear/Yellow/Green \| \| - Runny Nose \| - Headache \| \| - Blocked Nose \| - Fever \| \| - Wheeze \| - Muscle Ache \|   **Other:** | **How severe are the symptoms?** | | | | | | | | |
|  | \|  \| Severity \| \| \| \| \| --- \| --- \| --- \| --- \| --- \| \| Symptoms \| **No** \| **Mild** \| **Moderate** \| **Severe** \| \| Sneezing \|  \|  \|  \|  \| \| Itchy Nose \|  \|  \|  \|  \| \| Itchy Ears/Palate \|  \|  \|  \|  \| \| Runny Nose \|  \|  \|  \|  \| \| Blocked Nose \|  \|  \|  \|  \| \| Itchy Watery Eyes \|  \|  \|  \|  \| \| Headache \|  \|  \|  \|  \| \| Wheeze \|  \|  \|  \|  \| | | | | | | | | |
| **How often do these symptoms affect your sleep/performance/daily activities?**   \| **Weekly** \| \| --- \| \| **LESS** < 4 times/week > **MORE** \| \| **Yearly** \| \| **LESS** < 4 weeks/year > **MORE** \| | **How often do you use your medications to keep your symptoms under control?**   - Everyday - Other:______________ | | | | | | | | |
| **What brings on/makes your symptoms worse?**  **Is there, if any, a particular time of the year that this symptoms occur?** | | | | | | | | | |
